# Hospital and Institutional News

**Published:** 1918-06-22

**Authors:** 


### HOSPITAL AND INSTITUTIONAL NEWS.

### INCREASE IN PRICE—SPECIAL NOTICE.

IN consequence of the necessary economy in paper and the increasing costs of production it becomes essential *to raise the price of* THE HOSPITAL *to 2d. weekly*, commencing with the issue of Saturday, July 6, 1918. The paper question has continuously caused difficulties which have led to progressive increases in the cost of production and decreases in the supply of paper, and whilst these conditions prevail the price to the reader must inevitably be affected.

### AMERICAN WOUNDED AT PORTSMOUTH.

SINCE the American authorities, who have already generously allowed their troops in France to be brigaded with the soldiers of the Allies, are also anxious to make their medical and nursing staffs similarly available for the common cause, by which British staffs may be set free for work in other hospitals, two sections of a general hospital at Portsmouth have been officially examined. British soldiers, we understand, will not be ejected from the institution, but as vacancies occur the majority of patients admitted is likely to be American. Now that American wounded are being brought to England in the same convoys as British soldiers the matter should be easy to arrange. This information has been elicited from the War Office by Sir B. Falle, the Unionist Member for Portsmouth, whose wife, by the way, used to interest herself in the constituency on his behalf in many ways, and even ran a series of debates and addresses on general subjects which used to be attended by the wives of the electors. Now that the latter will have the vote, or many of them, the wives of members of Parliament will be encouraged to take an even more active interest in the women than they have done hitherto.

### A CANADIAN HOSPITAL FOUNDER.

WE were sorry to see the announcement of the death at Toronto of Mr. J. Ross Robertson, whose public activities far exceeded his positions as a newspaper proprietor and a prominent Canadian Freemason. He helped, more than anyone, to establish the Children's Hospital, Toronto, which he generously helped to build and no less generously to maintain. Its convalescent home was solely his gift, so was the hospital's nurses' home, which he not only built but furnished. A short account of his hospital work was given in THE HOSPITAL of May 7, 1910, from which we republish the following: "In 1883 the city of Toronto granted a plot of land upon an island in Lake Ontario, near the city, for the erection of a convalescent home. Mr. Robertson erected the first building; he added a wing in 1886; and in 1891 caused the entire building to be remodelled and the accommodation to be more than doubled at a cost of over $30,000 (£5,000)." He was a typical mainstay, and a characteristic product, of the voluntary system; a man of business who liked to place his experience, time, and knowledge at his hospital's disposal, and found, like many another, that this super-selfish work was its own reward, and gave to him the elation of escape from merely personal or business interests.

### THE DANGER OF UNQUALIFIED PERSONS ON HOSPITAL STAFFS.

IN our issue of April 27 we commented adversely on the fact, as revealed in the course of a coroner's inquest, that certain of the patients at the Royal South Hants and Southampton Hospital had been placed under the care of an unqualified person. Relative to this comment we have received a letter from Dr. Omar, who was, it seems, acting as house physician at the hospital at the date when the child whose death was the subject of official investigation was discharged. Dr. Omar, in his letter, contests the accuracy of the *post-mortem* conclusions, the finding of the jury, and "the rotten system of appointing coroners who are not medical men"—points on which our note expressed no opinion. But he does not challenge the evidence that the discharge of the child from the hospital was the act of a person who holds no medical qualification, and this alone was the motive of our censure. It well may be that in the present emergency certain hospitals are unable to obtain the services of a fully qualified resident staff, and have to be content with the help of senior medical students. But in such an event it is manifest that the resident staff ought not to be permitted to act except under the immediate responsibility and direction of some fully-qualified practitioner, and the discharge of a patient from a hospital, apart from this sanction, cannot therefore be justified. Dr. Omar, as we understand his position, does not challenge either the general principle which we now reaffirm, nor does he deny that in the case in question this principle was disregarded. The other questions raised in his letter doubtless have their importance, but they are not relevant to the issue which we discussed in our original note.

### LORD KNUTSFORD'S SERMON.

THE presence of lay speakers in churches is now established, as anyone who has watched the congregation at the weekday addresses given by clergy and others at St. Martin's-in-the-Fields is well aware. It was not a precedent, then, when Lord Knutsford ascended the pulpit of the Church of St. Philip, Whitechapel, last week, to give an address on Edith Cavell. Besides, this is known as the London Hospital Church. In reminding the congregation of Edith Cavell's connection with the nursing staff of the London Hospital the speaker said that her death was not the end, but rather the kindling of the light of her influence. It had made that light so to shine that men and women in every place had been led to glorify God. A careful summary of the events which led to her execution, which popular feeling is apt to confuse, was given in THE HOSPITAL of October 6, 1917, pp. 5 and 6.

### TOMBOLAS AND HOSPITAL ETHICS.

The old question how far the end of charity may be allowed to justify the employment of an appeal to the gambling instincts of mankind continues to crop up in connection with war charities. The Government, in the person of Mr. Brace, prefers, according to the English law of compromise, which is called Pecksniffian on the Continent, to wink at the practice rather than expressly either to allow or to forbid it. The Metropolitan Commissioner of Police is technically ready to pounce upon any tombola, lottery, or raffle, and Sir C. Henry has failed to obtain an undertaking that the Commissioner shall be instructed to waive his repressive action in respect of those undertaken for the benefit of war charities. In our view, it is absurd to take lotteries too seriously, for there is no deception of the public; but, in so far as voluntary hospitals are concerned, we regard recourse to them as unseemly, since a voluntary hospital has sufficient claims to attention to make it wholly independent of controversial methods of finance which are beneath the dignity of their charity.

### AMERICAN ASSISTANCE AT YORK.

The Archbishop of York, who has been taken to task for endeavouring to apply the Sermon on the Mount to the passions of patriotism, has done a good turn to the York County Hospital by handing to its committee £100 out of the funds placed at his disposal by the Emergency Aid British Relief Committee of Philadelphia to aid in the establishment of an orthopædic department. Accordingly, such a department is being set up at York County Hospital. This will add to the facilities already in existence by supplementing the treatment now available at Beckett's Park Hospital, Leeds. With this send-off from America there should be little difficulty in raising the remaining money required.

### A CONVALESCENT HOME FOR DISCHARGED MEN.

The discharge of a man from the Army is now at last seen to be not the end of our responsibilities towards him, but in many cases the beginning. Therefore the Birmingham Branch of the Red Cross Society has lately added to its bewildering list of activities for the wounded a convalescent home for discharged soldiers. Its object is to tide over the interval between nominal discharge from hospital and the real moment when recuperation is complete. It should provide as happy a time as the "after-cure" which rich people used to enjoy once their ingestion of distasteful waters was completed. The home is at Aberystwyth. No cases requiring medical treatment have hitherto bent sent—a salutary rule if the people for whom the home is intended are not to be crowded out by others who should never have been allowed their discharge from hospital or semi-convalescent home at all. To keep six men costs £9 a week. The space is available for twelve. The men are there. It only remains to provide the money. Mr. E. C. Thomas, the organising secretary, is not the man to despair.

### AMERICAN NAVY'S HOSPITAL IN LONDON.

Mrs. Guest, the wife of Capt. the Hon. Frederick Guest, M.P., has lent her town house, 26 Park Lane, which was built by Mr. Otto Beit, to the American Red Cross as a hospital for the American Navy. It will contain fifty beds. Mrs. Guest is the daughter of Mr. Phipps, of Pittsburg, and the loan of her house to the American Navy, therefore, is a patriotic one. The house will be known as American Red Cross Hospital Number 25.

### A RED CROSS RESTAURANT.

For the benefit of American officers who are attached to naval and military offices in London a restaurant has been opened by the American Red Cross at the United States' Army Headquarters, Grosvenor Gardens. The committee in charge is composed of English and American ladies, Lady Arthur Pearson and Mrs. Whitelaw Reid among them. The meals are served in what was the old dining-room of the Belgravia Mansions Hotel, the building, in fact, which was taken over for the U.S. Army Headquarters. Luncheons are served for half-a-crown, and dinners for three shillings and sixpence. The Red Cross, in short, will soon have to change its motto and adopt the familiar if humble claim of a well-known "emporium" by announcing itself to be "the Army's universal provider."

### THE SALARIES OF WOMEN SURGEONS.

The Elizabeth Garrett Anderson Hospital for Women has been advertising for two house surgeons and an obstetric assistant at a salary of £50 a year. Of course, board, residence, and laundry are included. This is a very humble salary to offer, and though candidates for women house surgeonships will often prefer to work at a hospital staffed by women, we should have thought that in these days a more adequate salary was required. Required or not, it would be certainly desirable. It is, however, only fair to quote the defence offered by Dr. Sybil Pratt, the hospital's house physician, who has written to a contemporary in the following terms: "Before the war," Dr. Pratt remarks, "most of the London hospitals regarded, and still regard, the experience gained as sufficient return for the services of the resident physicians or surgeons, whether men or women. I think I am right in saying that the E. G. A. Hospital was one of the first to pay its resident staff any honorarium at all. . . . The value of a resident post depends only secondarily upon the honorarium, and many old resident medical officers of this hospital will agree with me in saying that the training and experience gained while holding these posts are among the best investments they have ever made." This will be readily admitted, and when Dr. Pratt goes on to add, "I have no reason to suppose that, once the present urgent needs of endowment, upkeep, and enlargement are met, the hospital authorities would refuse to accept donations especially given for the increase of the salaries of their staff," the experienced administrator sees a good opportunity of exploiting among women sympathisers the popular cry "Equal pay for men and women." It is by no means certain that salaries on the modern scale should not come before the other great needs which Dr. Pratt mentions. A hospital must set an example as well as broken limbs: to maintain the highest standard, not only for its patients, but for its doctors', nurses' and domestics' well-being.

### A HOSPITAL EXPLOSION AT BIRMINGHAM.

JUST before nine o'clock at night an explosion occurred in the engine-room of the Birmingham General Hospital on Wednesday last week. The noise, which was heard in the street, led to the central fire station being informed, whereupon the chief officer, with the brigade and fire escape, together with the whole Lingard Street brigade, appeared on the scene. It was luckily found that there was no fire, but the wall of the engine-room had been blown out by an accumulation of hot air in the hot-air chamber. The momentary alarm did not reach the patients, who, in the large buildings, were mostly unaware of what had happened.

### "SACRILEGE" AVERTED AT A MILITARY HOSPITAL.

SOUTHWARK BOARD OF GUARDIANS have succeeded in their protest against the conversion of the chapel at their infirmary, which is now used as a military hospital, into a recreation-room for wounded soldiers. The facts were stated in the issue of THE HOSPITAL of May 23. The Guardians have been informed that a letter has been sent by Major-General S. Macdonald, D.D.M.S., London District, to Lieut.-Colonel Butler, who is in charge at the Southwark Military Hospital, stating that it had been decided that on no account must the chapel be interfered with, as the buildings were lent to the military authorities by the Guardians under certain conditions, and without their consent no alterations could be made. He asked that the chapel should be at once restored to its original condition. We welcome this return to common sense.

### THE SPANISH EPIDEMIC.

THE *Times* correspondent, writing from Madrid, gives particulars of the unknown disease which for the past three weeks has been ravaging the Iberian peninsula. In Madrid, he states, there are 100,000 victims alone, the tramway services are reduced and the telegraph disorganised; it has spread throughout Spain, and has even reached the sailors of the Fleet and the garrison in Morocco. At first its benign character led to its being the occasion for much humour in the press, but latterly it appears to have lost its innocent reputation, for 700 deaths are reported in the space of ten days, though most of these presented some complication. Dr. Pittaluga, writing in the *Sol*, describes the disease as "appearing suddenly without premonitory symptoms, accompanied by severe headaches during a few hours, high fever, throat irritation or slight bronchitis with a dry hacking cough and complete loss of appetite, muscular and articular pains, general debility, and gastric disturbance. The second day is marked by profuse perspiration, the fever diminishes, and disappears on the third or fourth day." The suggestion made in the *British Medical Journal* that the disease is gastro-intestinal influenza does not appear to have been borne out by bacteriological examination, for the influenza bacillus has not been isolated, but a parameningococcus has been shown to be present. Treatment is constitutional rather than medicinal, fresh air and good food being of greater service than drugs.

### A PROFESSORSHIP WITHOUT A TITLE.

SIR JAMES E. JONES, a governor of the Royal Schools for the Deaf and Dumb, Old Trafford, Manchester, has announced his intention to found a lectureship, or a professorship, for the education of the deaf in connection with the Victoria University of Manchester. To complete his scheme Sir James proposes to provide hospital accommodation near the Old Trafford Schools for the instruction of the art of teaching the deaf. Imagine such a chair in the older universities! Truly the new delight to break new ground. Who can supply a convenient designation of the new chair?

### M. DE LEVAL AND NURSE CAVELL.

AMONG the mass of names added to the enormous Order of the British Empire stands out that of M. Gaston de Leval, the distinguished member of the Belgian bar who gained the ear of Europe through his forensic efforts on behalf of his most famous client, Nurse Edith Cavell. M. de Leval is awarded the C.B.E., and his appointment confers no less honour upon the Order than his Companionship does upon himself. M. de Leval has also done useful work in the United States, and his official recognition in this country will be appreciated, we hope, as deeply among the Belgians as it is by ourselves.

### PRESENTATION TO A WOMAN HOSPITAL SECRETARY.

A PRESENTATION in the form of a cheque, a Treasury note, and a lady's leather suit-case has been made to Mrs. Fred Grace on her retirement from the post of secretary to the Keighley Victoria Hospital, in order to take up work at Coventry. As THE HOSPITAL has previously noted, Mrs. Grace has been a worthy successor to her father, and has spent eighteen years in hospital work at Keighley.

### A WOMAN MEDICAL OFFICER OF HEALTH.

DURING the absence of Dr. Oates on military service the Ilford Urban District Council has appointed Dr. Grace H. G. Dundas, medical officer, Child Welfare Centre, Leith, formerly deputy medical officer of health, and deputy school medical officer for the borough of Ramsgate, as medical officer of health, at a salary of £550 per annum.

### DEATH OF A PROMINENT PUBLIC PHARMACIST.

BY the death of Mr. William E. Miller public pharmacy loses a prominent worker for the good of pharmacists engaged in hospitals and similar institutions. Mr. Miller, who was a member of the Pharmaceutical Society, retired from his position as senior pharmacist to the St. Pancras Guardians some four years ago, and on that occasion received a valuable presentation from the officials, and in addition was granted the maximum pension allowable in recognition of his faithful services for over thirty years. Mr. Miller was one of the original founders of the Public Pharmacists' Association, and his residence at Clarendon Square was for many years the meeting-place of the Council. Much of the progress that has been made was due to his foresight, and on his retirement he was presented with a collection of books and elected an honorary member of the Association. Although retired on pension and quite entitled to spend the evening of his days quietly, Mr. Miller preferred to assist many institutions by acting as "locum," and thus enabling the pharmacist to secure a holiday. It was at Oxford, whilst acting in such a capacity, that Mr. Miller was taken suddenly ill on June 1, and passed away the same day at the age of sixty-nine.

### FARM COLONIES FOR CONSUMPTIVES.

At the Royal National Sanatorium for Consumption, Bournemouth, Mr. D. H. W. Robson-Burrows presiding, a further discussion has taken place on the proposed formation of a farm colony in association with the institution. Referring to the suggested schemes for placing discharged soldiers on the land, Mr. Child Clarke expressed the opinion that if the institution acquired fifty or sixty acres of land, and suitable patients were taught to cultivate it, they would make better landholders than many who took up the work without knowledge. He suggested that the matter be referred to the Government for their support. Dr. Hyla Groves, speaking from his experience of the Y.M.C.A. Farm Colony for Discharged Tuberculous Soldiers, warmly supported the suggestion. He considered that the ultimate solution of the treatment of consumptives lay in the formation of farm colonies which would probably become the nuclei of small communities engaged in occupations not confined to agriculture and gardening. The chairman expressed his appreciation of the remarks of the various speakers, and said that the committee would be glad to take every step in its power to see what could be done in the matter. Last year 575 patients were under treatment, when, indeed, all the beds were continuously occupied. The expenditure for the year amounted to £8,128, and the income (including £350 received from legacies) to £8,658. The patients included a considerable number of sailors and soldiers. Mr. Robson-Burrows expects in the near future to receive more patients discharged from the Services. The case for these farm colonies has been often advocated by enthusiasts in The Hospital, and we should like to see Mr. Clark's proposal put to the practical test for which he and Dr. Hyla Groves hope.

### A PROPOSED ASYLUMS CONFERENCE.

A conference of visiting committees of asylums has been proposed with a view to strengthening them in dealing more efficiently with the economical administration of asylums, and also with the permanent officialism of the Government departments, who, in the opinion of the committee of the Bucks County Asylum, will probably be the greatest opponents of reconstruction. As the Medico-Psychological Association has commenced a campaign for the amendment of all the Lunacy Acts this committee thinks the present a fitting opportunity to found a permanent method of co-operation among visiting committees of county and borough asylums in England and Wales.

### BIRMINGHAM'S NEW INFANT HOSPITAL.

In connection with the maternity and infant welfare work of the Birmingham Corporation the Health Committee has long desired to provide hospital treatment for infants. An opportunity has now occurred through the closing of an institution which was established at Barnt Green as a school for nurses. The committee of the institution, which is fully equipped, offers it for a hospital for sick infants sent by the Corporation on condition that the working costs are secured. The Health Committee accordingly proposes a grant of 25s. per week in respect of fifteen infants. About seventy children can be treated annually. It is proposed to try the experiment for a year.

### THIS WEEK'S DRUG MARKET.

Few noteworthy price changes have occurred this week, and the volume of business in the drug market has been comparatively small. The lower tendency in the price of phenacetin is still in evidence; the value of phenazone is barely maintained, and the same may be said of salicylic acid and salicylate of soda. In quinine a small business is passing at unchanged rates. The demand for bromides is quiet, pending the expected Government control of these drugs. Aspirin sells at steady rates. Antifebrin is still scarce, and the price is well maintained. An advance in the price of chloral hydrate seems probable. Cream of tartar is less scarce than it has been for some time past, and the value is not so firmly maintained. Lanoline is in good supply at lower rates. Buyers are disposed to pay more attention to menthol, the present price of which may be considered reasonable, in view of all the circumstances. Makers of lithia salts have raised their quotations. Iron and ammonium citrate, potassium citrate, and sodium citrate are dearer. Makers of belladonna extract have fixed the new season's price at a lower figure than that which was previously ruling. Henbane leaf is a little lower in price.

